# A “Sconce” Trap for Sampling Egg Masses of Spotted Lanternfly, *Lycorma delicatula*

**DOI:** 10.3390/insects16070689

**Published:** 2025-07-01

**Authors:** Sarah M. Devine, Everett G. Booth, Miriam F. Cooperband, Emily K. L. Franzen, Phillip A. Lewis, Kelly M. Murman, Joseph A. Francese

**Affiliations:** 1 Forest Pest Methods Laboratory, United States Department of Agriculture, Animal and Plant Health Inspection Service, Plant Protection and Quarantine, Science and Technology, Buzzards Bay, MA 02360, USA; 2 Department of Biology, Xavier University, Cincinnati, OH 45221, USA; 3 Bethel Field Station, United States Department of Agriculture, Animal and Plant Health Inspection Service, Plant Protection and Quarantine, Science and Technology, Bethel, OH 45106, USA

**Keywords:** invasive species, detection, egg mass trap, tree of heaven

## Abstract

Survey and detection of the spotted lanternfly, *Lycorma delicatula* (White), rely either on traps that exploit the insect’s behavior as it navigates its environment, or on visual surveys of either its mobile life stages or egg masses. A recently described egg mass trap, coined the “lampshade” trap, can assist with early detection in newly infested areas, provide egg masses for researchers, and potentially facilitate spotted lanternfly population reduction by removal of egg masses from the environment. Here, we describe a modified lampshade trap, the sconce trap, that uses less material, can be pre-cut prior to deployment, and can be deployed by one person, representing potential cost, labor, and time savings. Both traps were comparable at detecting populations of spotted lanternflies, and while females deposited more eggs on the larger lampshade traps, they deposited more eggs on sconce traps as a function of trap area.

## Simple Summary

While the “lampshade” trap designed for the collection of spotted lanternfly (*Lycorma delicatula*) egg masses is effective, we propose a similar, simpler design that uses less material and is easier to deploy. This “sconce” trap, when compared to the lampshade trap, had similar SLF detection rates and resulted in more egg masses per area of roofing material. While the larger lampshade traps can hold more egg masses per trap, more sconce traps could be constructed from the same amount of material, casting a wider, more cost-effective net for detection.

## 1. Introduction

Spotted lanternfly (SLF), *Lycorma delicatula* (White) (Hemiptera: Fulgoridae) has spread to 18 states across the eastern United States since its initial discovery in 2014 in Pennsylvania [1,2]. Native to Asia, this phloem-feeding fulgorid can adversely affect its 103 species of host plants, of which 56 species occur in the USA [3]. Although a generalist, its preferred host is tree of heaven (TOH), *Ailanthus altissima* (Miller) Swingle (Sapindales: Simaroubaceae), which is also widely invasive in the USA [2]. Survey and detection of spotted lanternfly rely on either traps that exploit the insect’s negative geotaxis as it walks up tree trunks [4,5] or visual surveys of mobile life stages as well as eggs [6]. Lewis et al. [7] describe a passive “lampshade” egg mass trap as attractive to female SLF for egg deposition. These traps were constructed from 22.86 cm wide roofing asphalt product placed around the circumference of a host tree. Two layers of fiber batting were attached to the trunk with cable ties near the top of the trap, followed by an inwardly facing length of the roofing asphalt attached by staples to create a sheltered area around the tree. This design resulted in an area where spotted lanternflies preferentially concentrated and deposited their egg masses. Egg masses from the traps could either then be destroyed or used in research. Additionally, collecting egg masses from these traps has proven to be easier and faster than searching for and chiseling them from bark surfaces.

While an excellent tool for egg mass collection, the lampshade trap uses a considerable amount of material that must be cut to size in the field, can be unwieldy to carry through field sites, and requires two people to install [7]. We compared egg mass collection of SLF on lampshade traps and on newly designed sconce traps. The “sconce” trap design alternatively utilizes a single, smaller pre-cut piece of the same roofing asphalt stapled to a tree and eschews the fiber batting. The sconce trap offers a cost-, time-, and labor-saving method while retaining the overall ability of the trap to collect and detect egg masses of SLF.

## 2. Materials and Methods

### 2.1. Egg Mass Traps

**Figure 1:**
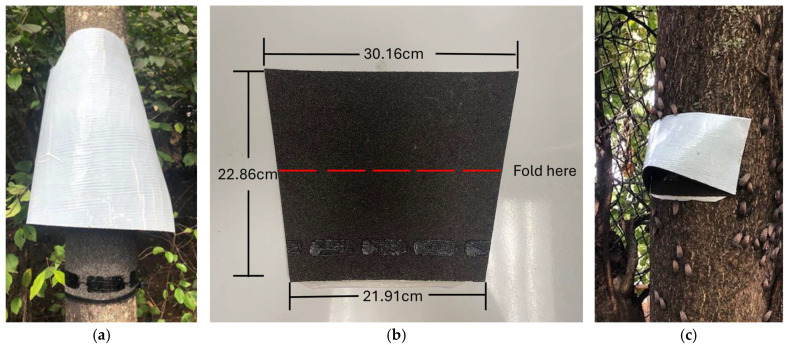
Egg mass traps including (**a**) a lampshade trap encircling the *A. altissima* tree, (**b**) a sconce trap secured on the SSW side of the host tree, and (**c**) a pre-cut sconce trap, prior to installation, showing di-mensions and the fold line. For additional sconce deployment information, an instructional video is provided in the Supplementary Material.

Two egg mass trap designs were compared. The “lampshade” trap was constructed as described by Lewis et al. [7] and following instructions provided by University of Massachusetts [8]. Lampshade traps were cut to encircle the host tree (*Ailanthus altissima*) placing a strip (22.86 cm wide) of roofing material (GAF Quick-start Starter Strip, Parsippany, NJ, USA) around the tree’s circumference, approximately 1.5 m above the ground, followed by two layers of fiber batting material (Fairfield, Danbury, CT, USA) plus an additional length of roofing material equaling the circumference plus 5.08 cm to create the outer covering (Figure 1a). Sconce traps used the same roofing material cut into 21.91 cm × 30.16 cm × 22.86 cm high trapezoids (595.16 cm^2^; Figure 1b), regardless of tree circumference. The material was then placed on the host tree, with the longer length on top, and folded along the latitudinal middle of the trap with the corners stapled to the tree (Figure 1c). All sconce traps were placed facing SSW to correspond to increased SLF egg mass distributions found in earlier surveys (KMM, unpublished data).

### 2.2. Trapping Assays

Thirty-four pairs of traps, treated as replicates, were placed in pairs on *A. altissima* of similar diameter at breast height (DBH; 19.3 ± 1.1 cm and 18.6 ± 1.2 cm for lampshades and sconces, respectively; Table 1). The pairs were spread over five counties in four states, MA (*n* = 10), NJ (*n* = 14), NY (*n* = 1), and OH (*n* = 9). Traps were placed in the field in mid-July 2024 prior to adult emergence. Trap pairs were removed from the field in winter 2025, following the end of the SLF adult flight and oviposition period [9] and taking into consideration any landowner restrictions on when traps could be harvested. During removal, the number of SLF egg masses on each trap surface (including those that came loose when taking the traps down) was recorded.

### 2.3. Statistical Analyses

The distributions of the data from the trapping assays were tested for normality in JMP 10 (SAS Institute 2012, Cary, NC, USA). The data were not normally distributed, so Wilcoxon tests of summed ranks (α = 0.05), blocking by replicate, were performed to determine if there were significant differences between trap types. A Fisher’s exact test was conducted (α = 0.05) to determine if the percentage of traps that had collected egg masses differed significantly between trap types. This test was performed using Excel for Microsoft 365 (2019).

## 3. Results

The average area of a lampshade trap was ~5.26 times larger than the area of a sconce trap (Table 1). Significantly more (Z = −6.80, df = 1, *p* < 0.0001) egg masses were deposited on lampshade traps than on sconce traps (Table 1). When egg mass deposition was compared as a function of trap area, significantly more (Z = 3.47, df = 1, *p* = 0.0005) egg masses were deposited per cm^2^ of roofing material on sconce traps than on lampshade traps (Table 1). Egg masses were deposited on 97.1% (33 out of 34) of lampshade traps and 94.1% (32 out of 34) of sconce traps (Table 1). These rates were not significantly different (*p* = 0.62). The three replicates where only one trap type collected egg masses were likely set in low-SLF-density areas, as evidenced by low trap catch (fewer than seven total egg masses). These represented three of the four lowest totals of egg masses collected between pairs, while 29 pairs collected at least 15 egg masses between both traps.

## 4. Discussion

In 32 of the 34 pairs, the number of egg masses collected was higher on the lampshade trap. This is not surprising, as the lampshade trap has a much larger surface area than the sconce trap and covers the entire circumference of the tree. The greater surface area and trapping ability of the lampshade trap allows researchers who need to rear SLF for studies to collect more egg masses with a single trap compared to the smaller sconce design.

However, there are several benefits to the sconce trap. Sconce traps can be cut prior to being taken to the field, and because of this, the amount of material needed can be estimated more effectively prior to deployment. Several manufacturers sell the material in 30 m rolls. Consequently, 99 sconce traps could be cut from one roll regardless of tree diameter, whereas for 19 cm diameter trees like those used in this study, only 22 lampshade traps could be cut from the same roll. Additionally, the fiber batting that holds the lampshade trap open is not necessary in the sconce trap and may hinder oviposition since few to no egg masses were found on the batting. The sconce trap may be more practical for very-large-diameter trees since it saves on material, is lighter to carry, and only requires a few staples by a single surveyor. The sconce trap is also more practical to use on trees whose full circumference is not fully accessible because of obstacles such as vines or fences, which can then be avoided. The versatility of the sconce trap also allows for multiple traps to be deployed on different sides of the same tree. Additionally, as detection tools, both traps were highly effective at detecting SLF populations (i.e., finding or collecting a single target insect or egg mass [10]).

These traps can be set prior to adult flight and only require a single check and disposal after the flight is over, representing cost, time, and labor savings for surveyors. Both trap designs effectively create an attractive oviposition site like the underside of branches or crooks in a tree where SLF frequently oviposit [7,11]. Lampshade and sconce traps can both be used to provide information on the presence of egg masses at a site, which can assist regulatory programs with strategies for upcoming field seasons.

## Figures and Tables

**Table 1:** A comparison of mean trap catch (±S.E.), trap catch as a function of trap surface area (±S.E) and percentage of traps collecting egg masses for two types of egg mass traps. Differences in catch between trap types are denoted by different letters.

Trap Type	*N*	Tree DBH (cm)	Mean No. of Egg Masses Collected Per Trap (±S.E.)	Mean Trap Surface Area (cm^2^) Per Trap (±S.E.) ^1^	Mean No. of Egg Masses Collected Per cm^2^ of Trap Surface (±S.E.)	Percentage of Traps Collecting Egg Masses
Lampshade	34	19.3 ± 1.1	46.6 ± 6.2 *a*	3132.1 ± 952.3	0.017 ± 0.002 *b*	97.1%
Sconce	34	18.6 ± 1.2	14.9 ± 2.2 *b*	595.2	0.025 ± 0.004 *a*	94.1%

^1^ Both trap types are constructed from 22.86 cm wide rolled asphalt roofing material. Lampshade traps are designed to wrap around the tree, and therefore, trap size and surface area are dependent on tree diameter. A tree circumference-long strip of asphalt (backing material facing outward from the tree) is wrapped around the tree, followed by a strip of fiber batting (7.62 cm) then placed along the top of that strip, with an additional strip of roofing material, which is 5.08 cm longer than the tree’s cir-cumference, placed over that with the asphalt facing inward. Sconce traps are cut as a uniform (21.91 cm × 30.16 cm × 22.86 cm high) trapezoid that is folded in half and stapled to the tree.

## Data Availability

The raw data supporting the conclusions of this article will be made available by the authors on request.

## Supplementary Materials

The following supporting information can be downloaded at https://www.mdpi.com/article/10.3390/insects16070689/s1, Video S1: Sconce trap installation instructions.

## Abbreviations

The following abbreviations are used in this manuscript:

SLF	Spotted lanternfly
DBH	Diameter at breast height
USDA	United States Department of Agriculture
APHIS	Animal and Plant Health Inspection Service
PPQ	Plant Protection and Quarantine
S&T	Science and Technology
FPML	Forest Pest Methods Laboratory
